# Meeting Report From “Correlates of Protection for Next Generation Influenza Vaccines: Lessons Learned From the COVID‐19 Pandemic”

**DOI:** 10.1111/irv.13314

**Published:** 2024-10-08

**Authors:** Florian Krammer, Jacqueline M. Katz, Othmar G. Engelhardt, Diane J. Post, Paul C. Roberts, Sheena G. Sullivan, S. Mark Tompkins, Christopher Chiu, Stacey Schultz‐Cherry, Rebecca Jane Cox

**Affiliations:** ^1^ Department of Microbiology Icahn School of Medicine at Mount Sinai New York New York USA; ^2^ Center for Vaccine Research and Pandemic Preparedness (C‐VaRPP) Icahn School of Medicine at Mount Sinai New York New York USA; ^3^ Department of Pathology, Molecular and Cell Based Medicine Icahn School of Medicine at Mount Sinai New York New York USA; ^4^ Ignaz Semmelweis Institute, Interuniversity Institute for Infection Research Medical University of Vienna Vienna Austria; ^5^ The Task Force for Global Health Decatur Georgia USA; ^6^ Science Research & Innovation Medicines and Healthcare products Regulatory Agency Potters Bar UK; ^7^ Division of Microbiology and Infectious Diseases National Institute of Allergy and Infectious Diseases, National Institutes of Health (DMID/NIAID/NIH) Rockville Maryland USA; ^8^ WHO Collaborating Centre for Reference and Research on Influenza, Royal Melbourne Hospital, and Department of Infectious Diseases University of Melbourne at the Peter Doherty Institute for Infection and Immunity Melbourne Australia; ^9^ Department of Epidemiology University of California, Los Angeles Los Angeles California USA; ^10^ Center for Vaccines and Immunology University of Georgia Athens Georgia USA; ^11^ Center for Influenza Disease and Emergence Response (CIDER) University of Georgia Athens Georgia USA; ^12^ Department of Infectious Diseases University of Georgia Athens Georgia USA; ^13^ Department of Infectious Diseases Imperial College London London UK; ^14^ Department of Host‐Microbe Interactions St Jude Children's Research Hospital Memphis Tennessee USA; ^15^ Influenza Centre, Department of Clinical Science University of Bergen Bergen Norway; ^16^ Department of Microbiology Haukeland University Hospital Bergen Norway

**Keywords:** correlates of protection (CoP), COVID‐19, influenza, influenza vaccine, mucosal immunity, SARS‐CoV‐2

## Abstract

**Background:**

This report summarizes the discussions and conclusions from the “Correlates of Protection for Next Generation Influenza Vaccines: Lessons Learned from the COVID‐19 Pandemic” meeting, which took place in Seattle, USA, from March 1, 2023, to March 3, 2023.

**Conclusions:**

Discussions around influenza virus correlates of protection and their use continued from where the discussion had been left off in 2019. While there was not much progress in the influenza field itself, many lessons learned during the coronavirus disease 2019 (COVID‐19) pandemic, especially the importance of mucosal immunity, were discussed and can directly be applied to influenza correlates of protection.

## 1 Introduction

The identification of new correlates of protection (CoP) against influenza virus disease is an important goal supporting the development of next generation influenza vaccines, particularly those that provide greater breadth and durability of protection. Indeed, the Influenza Vaccines R&D Roadmap (IVR) highlights the need for new and validated immune CoP to accelerate development of both improved seasonal influenza vaccines and those that may mitigate future influenza pandemics [1]. Currently, the most commonly used CoP, first defined in 1972 [2], is the hemagglutination inhibition (HI) titer, a measurement of antibodies that inhibit the virus hemagglutinin (HA) from binding to sialic acid (the virus's receptor) on N‐linked glycans on the surface of red blood cells causing hemagglutination. HI antibodies bind close to the HA receptor binding site (RBS) and sterically hinder interactions between the HA and sialic acid. The HI assay has long been a convenient surrogate for measurement of antibodies that neutralize virus infectivity; virus neutralization (VN) assays are more complex as they require actual infection of cultured cells and take multiple days to complete. Numerous studies have shown that an HI titer of 1:32/1:40 is associated with a 50% reduction of risk for symptomatic influenza [2, 3, 4, 5, 6], which has been very useful for accelerated vaccine licensure and seroprotection studies. However, several other immune responses are associated with protection against influenza, independent of the HI titer. These include antibodies to the second surface glycoprotein, the neuraminidase (NA, the receptor destroying enzyme of the virus) [7, 8, 9], neutralizing antibodies [9, 10], HA binding antibodies [3, 11, 12], CD4 T‐cells and CD8 T‐cells [13, 14, 15]. Antibody Fc‐mediated receptor functions correlate with protection by passive transfer of human sera in animal models of viral infection [16, 17]. However, many questions remain. It is unclear which of these immune responses play a mechanistic role in protection and which are surrogates that cocorrelate with a true mechanistic CoP [18, 19]. It is also not clear how much of a correlate that is measured in peripheral blood can be truly mechanistic, since influenza infection is typically initiated at mucosal surfaces of the upper respiratory tract. The immune milieu in this region is different from the peripheral blood [20] and likely much more important for initial protection from infection and symptomatic disease, while systemic immune responses likely play a bigger role in protection from lower respiratory tract infection and progression to severe disease. Furthermore, the influence of sex, gender, age, comorbidities and underlying conditions, viral subtype and strain differences as well as transmission modes on immune correlates and protective thresholds remain largely unknown.

During the coronavirus disease 2019 (COVID‐19) pandemic, many influenza research and clinical trials were put on hold or delayed as development of COVID‐19 vaccines became a priority. Therefore, there was a need to understand what progress had been made since the last Correlates of Protection meeting in early 2019 [21], as well as to learn from the knowledge gained in the development of vaccines against severe acute respiratory syndrome coronavirus 2 (SARS‐CoV‐2) and their implications for next‐generation influenza vaccine development. The “Correlates of Protection for Next Generation Influenza Vaccines: Lessons Learned from the COVID‐19 Pandemic” meeting, the third in a series of meetings coordinated by ISIRV, took place in Seattle, USA, in March 2023 (program in Figure S1). This manuscript provides highlights and discussion from the meeting.

## 2 Lessons Learned From the COVID‐19 Pandemic and New Covid‐19 Vaccine Platforms

The COVID‐19 pandemic saw the use of transformational vaccine technologies, which now can be applied to other public health threats including influenza. A key lesson learned from the COVID‐19 pandemic was that new vaccines could be created, developed and rolled out quickly. Sarah Gilbert (University of Oxford) described the vaccine developed with the ChAdOx1 platform, which was created and in human trials in just over 100 days. The vaccine was given emergency use authorization in the United Kingdom in under 1 year. An equitable access strategy provided rapid scale up for global vaccine manufacturing allowing distribution of vaccine to over 170 countries. Collaboration was key to these successes. Further investments are needed to increase sustainable vaccine manufacturing sites in some regions.

Cheryl Cohen (University of the Witwatersrand) highlighted the challenges associated with the COVID‐19 pandemic (and seasonal influenza) in low‐ and middle‐income countries. The generation of local surveillance data was critical to guide the pandemic response using existing epidemiologic and virologic surveillance platforms. African nations had the lowest COVID‐19 vaccine coverage rates globally highlighting the need for local vaccine production and usage of seasonal respiratory virus vaccines to improve future pandemic vaccine responses. Next‐generation vaccines should aim to reduce transmission, in addition to illness.

Human challenge studies can provide evidence of vaccine efficacy early in clinical development, aid in identification of immune factors associated with protection, and provide a means to evaluate virus transmission intervention strategies. Chris Chiu (Imperial College) described how the SARS‐CoV‐2 human challenge platform characterized a primary respiratory virus infection in seronegative adults [22], providing a model of immunity for emerging respiratory viruses. Using nasal viral load as a read‐out, abortive or transient infections were associated with detection of cross‐reactive T‐cells and IgM, while immediate immune cell recruitment after infection was associated with functional protection.

Miles Davenport (University of New South Wales) presented combined data from vaccine clinical trials, evaluation of serum antibodies in breakthrough symptomatic infections, and prophylactic and therapeutic passive antibody studies. Neutralizing antibodies (nAbs) were shown to both predict and mediate protection from symptomatic and severe SARS‐CoV‐2. In vaccinated persons, nAbs were reduced against variant SARS‐CoV‐2 strains suggesting that nAbs titers can predict population susceptibility to and impact of emergent variants.

## 3 Next Generation/Universal Influenza Vaccines

The development of improved seasonal, more durable and broadly protective, or truly universal influenza vaccines remains a high priority for public health. Florian Krammer (Mount Sinai) presented an overview of potential targets for universal and/or broadly protective influenza vaccines. These include internal virus proteins such as nucleoprotein (NP), matrix protein (M1), and polymerase subunits; the role of antibodies targeting these antigens in protection is unclear [23], but specific T‐cell responses have been shown to be protective [14]. Targets that are more easily accessed by antibodies are the external domain of the M2 protein (M2e), the NA and conserved parts of the HA. Studies have shown potential for all of these, with antibodies against the NA and the HA stalk domain reported as independent CoP [3, 7, 8, 9, 12]. Various approaches to induce antibodies or increase antibody levels are being investigated. These include sequential heterologous vaccination; epitope dilution [24, 25]; display of individual epitopes; the use of consensus or ancestral antigens; and the freeing up of conserved epitopes or masking of immunodominant but variable epitopes (reviewed in [26]).

Aaron Schmidt (Harvard Medical School) focused on novel ways to present the conserved RBS of the HA to the immune system. The RBS of a seasonal H1N1 virus was grafted on to HA proteins from subtypes that have not circulated among humans as scaffolds. This approach resulted in increased breadth of the anti‐H1 HA response in a mouse model, but not increased frequency of anti‐RBS antibodies. However, expansion of RBS‐specific B‐cells was achieved when the same RBS was expressed in the context of a heterotrimer of three different HA head domains that were covalently linked.

Aubree Gordon (University of Michigan) described observational studies as tools in the search for CoP. These include cohort and transmission studies as well as hybrid studies whereby transmission studies are embedded within cohort studies. Such work has revealed HI and anti‐HA stalk antibodies as independent CoP [3], with NA‐inhibiting (NI) antibodies being another independent correlate for H3N2 and a marker for shorter disease duration in H1N1pdm09 infection [27].

Arnold Monto (University of Michigan) built on experience from and critique of past work on CoP for currently licensed influenza vaccines to make recommendations for similar work for new vaccines. Although seroprotection and the related measure of seroconversion, based on HI titers, have been used widely as CoP, they do not correlate well with efficacy of all influenza vaccines in all populations. For studies to define new CoP, reasonable numbers of subjects whose immune responses are measured are required; perhaps counter‐intuitively, very effective vaccines will require larger numbers, as vaccine failures (cases) are required for this analysis, and these will be rare for highly effective vaccines. Clinical and laboratory outcomes need to be related to potential predictors.

## 4 B‐ and T‐Cell Responses

To move towards prediction of protective immunity of SARS‐CoV‐2 and influenza vaccines, a better understanding of the components of innate, B‐cell and T‐cell immunity and the role they play in protective mechanisms is critical. Antibody binding and neutralizing activity are primarily evaluated to predict protection; however, non‐neutralizing antibodies can also protect against influenza virus infection [28]. Systems' serology approaches can be utilized to better understand non‐neutralizing functional antibodies and their role in protection against influenza. Galit Alter (Harvard Medical School) provided an overview describing how HI and neutralizing activity did not fully predict protection and that natural killer (NK) cell function has been linked to protection for influenza. Vaccine strategies able to leverage both neutralizing and non‐neutralizing antibodies are likely to confer the greatest level of protection. Determining mechanisms to enhance non‐neutralizing antibody activity, such as changing glycosylation of antigens and route of administration of vaccines, can be used to make more effective vaccines that can result in better NK cell activity.

Significant advances have been made to better understand the role of B‐cells in protection after influenza vaccination. Ultrasound‐guided fine needle aspiration has enabled the sampling of lymph nodes providing good representations of cell populations from this site [29]. Ali Ellebedy (Washington University in St. Louis) described how his laboratory is tracking B‐cell clones over time in blood and lymph nodes after influenza vaccination. They have shown that influenza vaccination can elicit a germinal center (GC) reaction that recruits B‐cell clones that can target new epitopes [30]. SARS‐CoV‐2 infections resulted in robust and persistent bone marrow plasma cell responses that were antigen specific and long lived [31]. A persistent GC results in better memory and higher bone marrow plasma cells.

T‐cells can influence B‐cell dominance, and helping B‐cells and antibody responses is a major function of CD4 T‐cells. There have been substantial advances in the understanding of Tfh cells which are a specialized subset of CD4+ cells needed for GC and B‐cell responses [32]. Paul Thomas (St Judes Children Research Hospital) provided an overview on the markers and variables in T‐cells that are the most important for predicting a protective signature. In an evaluation of several cohorts, they found a consistent pattern that CD4 T follicular (Tfh) cells > NK cells > CD8 cells were the most important [33]. He also discussed that inducible costimulatory (ICOS) positive Tfh cells specifically are the most important determinant and may be important as a mucosal correlate of protection. When looking at COVID‐19 vaccination responses, he presented that robust Tfh cell responses play an important role in establishing long‐term immunity [34].

Shane Crotty (La Jolla Institute for Immunology) reminded us that multiple factors play a role in immunity against influenza virus and SARS‐CoV‐2. Immunological memory can consist of memory B‐cells, antibodies, memory CD4+ T‐cells, and/or memory CD8+ T‐cells [35]. By better understanding the kinetics of immune memory, vaccine design can be improved resulting in enhanced immune protection. For example, changing the kinetics of antigen delivery (by providing a slow delivery) resulted in an increased peak of autologous neutralizing antibody titers and can substantially augment GC activity in response to immunization [36].

## 5 Mucosal Correlates of Immunity and Protection

The identification and validation of mucosal immune CoP against influenza remain challenging. Peter Openshaw (Imperial College) discussed the importance of defining the parameters of protection of a CoP, be it protection from infection, transmission, hospitalization, severe disease, or death. There is a need for standardized reproducible and repeated sampling methods of the upper and lower respiratory tract to study mucosal immune responses. A nasal absorption device using synthetic absorptive matrices to absorb the mucosal lining fluid [37] was used to show that live attenuated influenza vaccine (LAIV) challenge in adults often induces either systemic IgG or nasal IgA. Early mucosal interleukin 33 (IL‐33) increased up to 8 h, much earlier than other cytokines and chemokines and returned to baseline by 24 h, and potentially impacts viral shedding [38].

Kanta Subbarao (WHO Collaborating Centre for Reference and Research on Influenza) reviewed older LAIV studies, reminding us that at suitable antibody titers, either systemic (serum HI or NI antibodies) or local nasal wash IgA can mediate protection from influenza [39, 40, 41]. Nasal wash IgA correlates with HI antibodies after LAIV vaccination in children [41], although there are problems with reliable collection of adequate quality and volume of nasal washes. LAIV induces local inflammation in the nose [42], with upregulation of B‐ and T‐cell genes [43] and Tfh cells in the draining tonsillar tissue 6–7 days after vaccination [43, 44]. LAIV induces better T_fh_ cell responses in the tonsils and systemic antibody responses in children with lower local salivary IgA levels [45]. LAIV induces mainly IgM in tonsils of unprimed children but durable memory B‐cell responses in blood, which are not boosted in primed children [46]. Overall, serum antibody is not a reliable correlate of immunity for seasonal LAIV, which induces a multifaceted response. Despite no detectable serum HI antibodies after vaccination with prepandemic H5 or H7 LAIV, the vaccines primed for a rapid robust HI antibody response after boosting with inactivated prepandemic influenza vaccine doses. After prepandemic LAIV vaccination of African green monkeys, H5‐specific plasmablasts were not found in the blood but in the mediastinal lymph nodes. Boosting with inactivated vaccine expanded primed H5‐specific GC and non‐GC B‐cells in the local axillary lymph node and the peripheral blood [47].

Rebecca Cox (University of Bergen) focused on local responses induced by vaccination in children and adults. High levels of influenza‐specific antibody secreting cells (ASC) are found in the nasal mucosal tissue in adults, which are not boosted by parenteral vaccination [48]. Influenza virus–specific ASC are induced in adults and primed children 7 days after parenteral influenza vaccination together with transient IgA in saliva [48, 49]. Previous influenza virus infection is important for induction of tonsillar IgG, IgA, and IgM ASC in children after inactivated vaccination, whereas IgM is only induced in unprimed children with a slower time course.

Mucosal antibodies can neutralize respiratory viruses providing protection at the portal of entry. Jennifer Gommerman (University of Toronto) reported that after SARS‐CoV‐2 infection, salivary IgG and IgA correlated with serum IgG and IgA up to 120 days postinfection [50]. Strong nasal IgA responses were observed after SARS‐CoV‐2 infection but not COVID‐19 vaccination in naive individuals, which waned after 6–9 months [51]. Nasal IgG mirrored serum nAbs. In previously infected individuals, mRNA COVID‐19 vaccines induced transient salivary mucosal IgA post first dose, which was not boosted by additional vaccinations and even declined in some individuals [52, 53, 54]. After two COVID‐19 vaccinations, subjects with lower serum and mucosal spike or receptor binding domain (RBD) specific IgA experienced variant breakthrough infection [52, 55]. Breakthrough infection with Omicron increased salivary IgG and IgA to the Omicron variant and ancestral SARS‐CoV‐2 (e.g., [54]).

Stacey Schultz‐Cherry (St Jude Children's Research Hospital) reported that both viral and host factors (such as sex, gender, comorbidities, malnourishment, ageing, obesity, and pregnancy) determine the extent and severity of virus‐induced lung damage after influenza and SARS‐CoV‐2 infection. Furthermore, there are differences in systemic and nasal cytokine levels after infection in obese versus lean subjects [56]. Overall, the session highlighted the importance of understanding mucosal immune responses and the need for improved mucosal vaccines to provide local immunity.

## 6 Lessons From International Consortia on Immunological Assays

Harmonization and standardization of immunological assays are key steps towards the identification and validation of CoP. David Montefiore (Duke University) provided lessons learned from clinical studies of human immunodeficiency virus 1 (HIV‐1) and SARS‐CoV‐2 in establishing neutralizing antibodies as immune CoP. The ideal requirements for standardized laboratory assays are high throughput, low cost, easily transferable between laboratories and assays, which can be validated. For neutralization assays, it is important to consider the types of cells used, both as targets for infection and cells for virus production. In 2005, an HIV‐1 env‐pseudotyped VN assay was formally validated and transferred to global laboratories with reference virus strains for production of standardized datasets and an international proficiency testing program. Neutralizing antibodies were inversely associated with COVID‐19 risk and directly associated with vaccine efficacy [57]. The SARS‐CoV‐2 nAb concordance survey (SNACS) found ~50‐fold difference in 50% inhibitory dilution (ID_50_) neutralization titers between SARS‐CoV‐2 live and pseudotyped virus entry inhibition assays. Overall, there was greater concordance for D614G variant SARS‐CoV‐2 neutralization titers among pseudotyped virus entry inhibition assays than live virus assays. However, the calibration factor used for the SARS‐CoV‐2 D614G virus did not apply to the Omicron variants, highlighting the challenge associated with assay standardization of variant viruses.

The next three talks focused on the FLUCOP (https://flucop.eu) project, which was an EU Innovative Medicines Initiative (IMI) private–public partnership aimed at standardization of assays for assessing influenza CoP to better evaluate seasonal human influenza vaccines. The primary goal of FLUCOP was standardization of the HI and microneutralization (MN) assays.

Othmar Engelhardt (Medicines and Healthcare products Regulatory Agency) presented harmonization and standardization of the HI assay using experimental and data‐driven processes. FLUCOP developed a consensus protocol, by sharing of protocols and identifying the different parameters for testing, to evaluate interlaboratory variation. The use of biological standards reduced interlaboratory variability compared to in house testing, with a matched antigenic pool giving the best improvement, indicating a pooled serum standard could have a substantial lifespan [58]. FLUCOP consensus protocols were tested in large collaborative studies using egg‐ and cell‐derived viruses, human serum samples, and standards. Strict harmonization improved interlaboratory variability with common source antigen for both influenza A virus subtypes and influenza B lineages (https://figshare.com/articles/media/FLUCOP_Training_module/14822475). Overall, human serum pools as biological standards for in‐house testing are as good at reducing interlaboratory variation as the use of consensus protocols and common antigens [58]. A similar process was used to develop the MN (short form) and VN (long form) assay protocols. There was a good correlation between MN and VN assays for all antigens, although direct comparison between neutralization titers was not possible. Human serum pools used as biological standards had a significant effect on reducing interlaboratory variability [58]. Group 1 HA stalk antibodies are broadly cross‐reactive within and between influenza A subtypes. An antibody standard from high titer group 1 HA stalk antibodies was produced and tested in 10 laboratories, which used in‐house assays to measure anti‐stalk antibodies by either neutralization assays or binding enzyme‐linked immunosorption assays (ELISA). The standard reduced interlaboratory variation in ELISA in most laboratories [59], but data were insufficient to evaluate the neutralization assays.

Emanuele Montomoli (University of Siena) presented work on validation of assays for measuring neuraminidase antibodies. A number of different assays are available for measuring anti‐neuraminidase antibodies including the methylumbelliferyl‐*N*‐acetyl‐neuraminic acid (MU‐NANA) assay, plaque size reduction in cell culture, accelerated viral inhibition assay (“AVINA”), ELISA using immobilized NA‐protein, and the enzyme‐linked lectin assay (ELLA) [60]. The ELLA was chosen for measuring NI titers, with the source of antigen identified as a critical reagent as anti‐HA antibodies interfere with measurement of NI antibodies. A multilaboratory collaborative study showed that the consensus ELLA‐NI protocol had consistent precision, linearity, and robustness using an N1 antigen, with a calibrator significantly improving interlaboratory agreement [61].

The FLUCOP consortium also developed protocols for standardization of cell‐mediated immune (CMI) assays. Gwenn Waerlop (Ghent University) presented efforts on harmonization of in‐house CMI assays with different reagents and equipment hampering data comparability and reproducibility. Pilot studies demonstrated interferon γ (IFN‐γ) ELISpot, and intracellular cytokine staining (ICS) in‐house procedures were highly diverse generating barely comparable results. Potential critical parameters were identified to create harmonized protocols; the assays were qualified including theoretical and practical training for both the IFN‐γ ELISpot [62] and the ICS [63] assays. The proficiency test of harmonized protocols decreased IFN‐γ variations by approximately 50% postharmonization, resulting in highly comparable interlaboratory data [62]. Clear correlation was observed between the two methods, although they cannot be considered interchangeable [62]. However, more efforts are needed to harmonize the ICS assay, such as alignment in flow cytometers, devices, gating templates or use of automated gating. Overall FLUCOP demonstrated that harmonization can substantially improve the comparability of data generated in a multilaboratory setting.

## 7 Regulatory Aspects of Next Generation/Universal Vaccines

Understanding and navigating the regulatory requirements for licensure remain challenging for many next‐generation vaccine developers. David Vaughn from the Bill and Melinda Gates Foundation reminded us of the tight timelines that all influenza vaccine manufacturers face during the annual vaccine update for seasonal vaccines. He also noted the Traditional and Accelerated Approval regulatory pathways used in the United States for licensure of seasonal vaccines, as well as the process that has been used for approval of pandemic vaccines. Jerry Weir from the US Food and Drug Administration (FDA) elaborated further on some of these regulatory issues, emphasizing that clinical development and evaluation of next generation influenza vaccines would follow the established regulatory pathways. He further noted that while a CoP is not required for licensure, relevant immunogenicity is critical for vaccine development and evaluation and in fact a surrogate endpoint that is “reasonably likely to predict” clinical benefit is necessary for the Accelerated Approval pathway. Marco Cavaleri from the European Medicines Agency (EMA) provided a European regulatory perspective on how CoP can facilitate the regulatory process for licensure of new vaccines. He described some of the ways in which relevant biomarkers are derived, how CoPs are defined, and how the design of pivotal clinical trials is influenced by the availability of an established correlate versus an immune marker that is suitable to infer protection. The remaining panel members including the session co‐Chair Chris Roberts (NIH), Sarah Gilbert, and Chris Chiu commented on regulatory lessons learned with the COVID‐19 vaccine platforms, the utility of human challenge studies to inform clinical development, and the need to conduct well‐designed clinical studies to interrogate the immune response elicited by vaccines. Considerable discussion was devoted to the complexity of CoP analyses that may encompass multiple immune effectors and may also be platform and/or target population specific (i.e., based on vaccine antigen, or age or immune status of the targeted population). To move next‐generation vaccines forward, sponsors will likely have to demonstrate non‐inferiority to current seasonal influenza vaccines and ideally also need to demonstrate increased breadth or durability. There was further discussion about how sponsors can demonstrate that a particular biomarker is appropriate for evaluation of a next‐generation vaccine and is “reasonably likely to predict” clinical benefit. The unmet need to establish a panel of viruses to demonstrate breadth and identification of the parameters to assess and define durability was also highlighted, which hopefully will be topics for a future workshop.

## 8 Epidemiology and Study Design

An overview of vaccine effectiveness studies was presented by Sheena Sullivan (Peter Doherty Institute for Infection and Immunity), which summarized common study designs, notably the test‐negative design (TND), for monitoring the real‐world effectiveness of vaccines and the suitability of these designs for identifying reliable measures of immune responses that mediate the protective effect of vaccines (mediators of protection; MoP). Peter Gilbert (Fred Hutchinson Cancer Center) shared his work on measuring antibody CoP for COVID‐19, using data from the Moderna mRNA vaccine trials [57]. He also proposed a modification of the TND for collecting data for CoP analysis. Finally, Miles Davenport presented his work on predicting the risk of infection and severe disease based on postvaccination nAb titers [64, 65]. He highlighted the fact that SARS‐CoV‐2 nAb was a very solid CoP because it was established in a previously naïve population. To establish a strong and consistent CoP in a population with prior exposure will be more challenging as has been shown for the influenza HI CoP.

The feasibility of designs for measuring CoP to identify an ideal design was discussed. The model suggested by Peter Gilbert to embed immunological studies in TND studies, already implemented by the US influenza vaccine effectiveness network (led by the US CDC), was discussed, with pros and cons evaluated. The discussion also touched on the use of prospective and likely very expensive cohort studies to better understand CoPs, the need to study different populations and age groups, the need for assessment of different types of correlates (drawing on earlier sessions about mucosal correlates and B‐ and T‐cell responses), and the limitations of those approaches both in terms of feasibility as well as reliability (drawing on learnings from international consortia for aligning immunological assays). Finally, the conflict between identifying a true MoP that requires performance of intense immunological assays and having a simple correlate that is amenable to high throughput was discussed. However, it was generally agreed that serial high‐quality serum, mucosal and peripheral blood sample collection was an important component of an optimal study design.

A series of short abstracts was selected aimed at understanding immune correlates to influenza and COVID‐19 vaccines, describing susceptibility to influenza virus and SARS‐CoV‐2 infection, virus shedding dynamics and transmission in experimental human challenge, therapeutic human antibodies, and immune profiles of SARS‐CoV‐2 and influenza virus infection. Early in the meeting, it was noted that correlates will vary by vaccine platform, antigenic target, and across the populations studied. The establishment and maintenance of cohort studies throughout the SARS‐CoV‐2 pandemic were essential and provided critical reagents and data with far reaching significance. Given the complex immunity landscape, an ever‐evolving pathogen and differences in intervention strategies, longitudinal cohorts provided a framework to move towards understanding CoP. Infection induced neutralizing antibody titers correlated with protection against reinfections; however, the protection was dependent on the degree of immune escape in the circulating variant compared to the strain that caused the prior infection. In addition, protection was observed even in the absence of detectable neutralizing antibody titers, pointing to additional immunity factors contributing to protection. Detailed data sets are required to evaluate population‐level immunity in the context of complex exposure histories.

The sentiment that we can induce broader protection and generate enhanced vaccines by understanding and targeting the appropriate immune responses came up frequently during the meeting. One example with a focus on influenza was to focus the immune response on the influenza virus HA stalk region to provide a more broadly protective vaccine. SARS‐CoV‐2 spike and non‐spike antibodies with Fc effector function, antibody avidity, CD4+, and CD8+ T‐cell responses were alternate correlates that play a critical role in recognition of variants, protection against severe disease and viral clearance.

## 9 Discussion and Summary

An important discussion point throughout the meeting was how CoPs and immune markers are used in the development of vaccines. HI titers can be used for accelerated approval of influenza vaccines in the United States, and CoPs are extremely useful from a regulatory perspective when expanding vaccine licensure into new populations or when changing vaccine formulations. However, for consideration by regulatory agencies, novel CoPs will need to be measured with high quality assays and linked to outcomes in clinical trials. There is growing recognition that multiple immune mechanisms contribute to inhibit viral infection, replication or spread to provide “layered” levels of protection. Therefore, there is a need for collaborative clinical studies that collect serial serum and mucosal samples and peripheral blood to evaluate the full spectrum of immune responses that may confer protection. CoP discovered by academic laboratories, for example, in cohort studies cannot automatically be used for regulatory purposes, creating a disconnect between regulatory agencies/vaccine developers and academic research. Bridging this disconnect in the future with additional dialogue and more harmonized and standardized assays may help to accelerate vaccine development and licensing. Another consideration is practicality. While academics, in search of a true CoP, are relying on more and more sophisticated methods and sometimes invasive sampling, such methods are impractical to establish a CoP in a Phase 3 vaccine trial with thousands or ten thousand of participants. Robust, reproducible, and ideally high throughput assays using samples collected through minimally invasive techniques are needed to propel vaccine development forward. Importantly, influenza vaccines can be (and have been) licensed without a CoP based on efficacy data alone. However, CoPs facilitate many aspects of vaccine development and may de‐risk investments.

Another extensive discussion point at the meeting was lessons learned from SARS‐CoV‐2 and COVID‐19 vaccines [66]. The timely development of these vaccines was a result of collaboration among multiple groups and between vaccine developers and regulators. Beautiful data by many different groups established that binding and neutralizing antibodies are CoP, especially early in the pandemic [57, 67, 68, 69, 70, 71]. nAb titers have been used globally, including in Europe (but not in the United States) for approval of novel COVID‐19 vaccines. However, another important message from SARS‐CoV‐2 is that mucosal immunity, and especially secretory IgA in the upper respiratory tract, is likely an important mechanistic CoP from infection [52, 55, 72]. The idea that injected vaccines would protect from SARS‐CoV‐2 infection was questioned early, and in fact, animal studies with many vaccine candidates showed great protection of the lower respiratory tract, but little protection of the upper respiratory tract [73]. Moving forward, the role of mucosal immunity in protection, highlighted for SARS‐CoV‐2, should be a strong focus of work on CoPs for influenza infections. Harmonization of mucosal sample collection and assay methods is needed and could be achieved through a consortium approach like FLUCOP.

While research on influenza CoP slowed during the COVID‐19 pandemic, this meeting provided a forum for the influenza vaccine research and development community to recapitulate what is known and new in the field of research, while focusing attention on critical gaps. Indeed, several of the IVR milestones were addressed at the meeting and included not only those for immunology and CoPs but also milestones that addressed regulatory challenges. Insights from COVID‐19 helped to gain new perspective on what is important moving forward, with better understanding of what might protect us from infection, symptomatic infection, and severe disease caused by influenza A and B infections. To implement this knowledge into vaccine design and to accelerate vaccine development, continued dialogue between the academic research community, vaccine developers, and regulatory agencies is needed.

## Author Contributions

**Florian Krammer:** writing–original draft, writing–review and editing, conceptualization. **Jacqueline M. Katz:** writing–review and editing, funding acquisition, conceptualization, writing–original draft. **Othmar G. Engelhardt:** writing–review and editing, conceptualization, writing–original draft. **Diane J. Post:** conceptualization, writing–original draft, writing–review and editing. **Paul C. Roberts:** writing–original draft, writing–review and editing. **Sheena G. Sullivan:** writing–review and editing, writing–original draft. **S. Mark Tompkins:** writing–original draft, writing–review and editing, conceptualization. **Christopher Chiu:** writing–review and editing, writing–original draft. **Stacey Schultz‐Cherry:** writing–review and editing, writing–original draft. **Rebecca Jane Cox:** writing–original draft, writing–review and editing, conceptualization, funding acquisition, resources.

## Conflicts of Interest

The Icahn School of Medicine at Mount Sinai has filed patent applications relating to influenza virus vaccine and therapeutics, SARS‐CoV‐2 serological assays, and NDV‐based SARS‐CoV‐2 vaccines, which list Florian Krammer as co‐inventor. Mount Sinai has spun out a company, Kantaro, to market serological tests for SARS‐CoV‐2. Dr. Krammer has consulted for Merck, Seqirus, CureVac, and Pfizer in the past and is currently consulting for Pfizer, 3rd Rock Ventures, GSK, and Avimex, and he is a cofounder and scientific advisory board member of CastleVax. The Krammer laboratory has also collaborated with Pfizer on animal models for SARS‐CoV‐2 and with GSK on influenza virus vaccine development in the past and is currently collaborating with Dynavax on influenza virus vaccine development. S.G.S. has consulted for Novavax, CSL Seqirus, Evo Health, Moderna, and Pfizer.

10

### Peer Review

The peer review history for this article is available at https://www.webofscience.com/api/gateway/wos/peer‐review/10.1111/irv.13314.

## Supporting information


**Figure S1.** The meeting program from the Correlates of Protection for Next Generation Influenza Vaccines: Lessons Learned from the COVID‐19 Pandemic.
