# Supplementary material for: Meeting Report From “Correlates of Protection for Next Generation Influenza Vaccines: Lessons Learned From the COVID‐19 Pandemic”
Source: Influenza Other Respir Viruses. 2024 Oct 8;18(10):e13314. doi: 10.1111/irv.13314 (PMC11461279; doi:10.1111/irv.13314)
Supplement: Supplementary file 1 — Figure S1. The meeting program from the Correlates of Protection for Next Generation Influenza Vaccines: Lessons Learned from the COVID‐19 Pandemic. [file IRV-18-e13314-s001.pdf]

Programme

Correlates of Protection for Next Generation Influenza Vaccines: Lessons Learned from the COVID Pandemic

1 - 3 March 2023 Seattle | USA

Day 1 Wednesday 1 March

Time	Programme
08:00	Registration / Poster Mounting
09:00	**Welcome and Opening Remarks** **Keith Klugman** Bill & Melinda Gates Foundation, USA
Session A	**Lessons Learned from the COVID-19 Pandemic and New COVID-19 Vaccine Platforms**<br>Chairs: **Jacqueline Katz** The Taskforce for Global Health, USA **Ros Hollingsworth** Bill & Melinda Gates Foundation, USA
09:05	**Lessons Learned from New COVID Vaccines**<br>**Sarah Gilbert** University of Oxford, UK
09:35	**Lessons Learned from the COVID Pandemic**<br>**Cheryl Cohen** University of Witwatersrand, South Africa
09:55	**What Can We Learn from the Human Challenge of SARS-CoV-2 and Influenza?**<br>**Chris Chiu** Imperial College London, UK
10:20	**SARS-CoV-2 Correlates of Protection**<br>**Miles Davenport** Kirby Institute, UNSW Sydney, Australia

**10:40**	**BREAK**
**Session B**	**Next Generation/Universal Influenza Vaccine**<br>Chairs: **Othmar Engelhardt** Medicines and Healthcare Products Regulatory Agency (MHRA), UK<br>**Kanta Subbarao** WHO Collaborating Centre for Reference and Research on Influenza, Peter Doherty Institute for Infection and Immunity, Australia
**11:10**	**Universal Influenza Vaccine**<br>**Florian Krammer** Icahn School of Medicine at Mount Sinai, USA
**11:40**	**Next Generation Influenza Vaccines**<br>**Aaron Schmidt** Harvard Medical School; Ragon Institute of MGH, MIT and Harvard, USA
**12:00**	**Correlates of Protection from Cohort Studies to Guide Next Generation Influenza Vaccine**<br>**Aubree Gordon** University of Michigan, USA
**12:20**	**Correlates of Protection for Next Generation/Universal Influenza Vaccine**<br>**Arnold Monto** University of Michigan, USA
**12:40**	**LUNCH**
**Session C**	**Mucosal Correlates of Immunity and Protection**<br>Chairs: **Ali Ellebedy** Washington University School of Medicine, USA **Rebecca Jane Cox** University of Bergen, Norway
**14:00**	**Mucosal Correlates of Immunity and Protection**<br>**Peter Openshaw** Imperial College London, UK
**14:30**	**Mucosal Data from LAIV**<br>**Kanta Subbarao** WHO Collaborating Centre for Reference and Research on Influenza, Peter Doherty Institute for Infection and Immunity, Australia
**15:00**	**The Role of Mucosal Immunity to Influenza**<br>**Stacey Schultz-Cherry** St Jude Children’s Research Hospital, USA

**15:20**	**Local Immunity After Influenza Vaccination**<br>**Rebecca Jane Cox** University of Bergen, Norway
**15:40**	**The Role of Local Antibodies in SARS CoV-2 Protection**<br>**Jennifer Gommerman** University of Toronto, Canada
**16:00**	**BREAK**
**Session D**	**Immunology: B and T Cell Responses**<br>**Chairs: Florian Krammer** Icahn School of Medicine at Mount Sinai, USA<br>**Diane Post** National Institute of Allergy and Infectious Diseases (NIAID), USA
**16:30**	**Role of Non HI Antibodies in Protection from Influenza**<br>**Galit Alter** Harvard Medical School, USA
**17:00**	**Role of B Cells in Protection after Influenza Vaccination**<br>**Ali Ellebedy** Washington University School of Medicine, USA
**17:30**	**CD8 T Cells in Protection from Influenza**<br>**Paul Thomas** St. Jude Children's Research Hospital, USA
**18:00**	**Role of T Cells in Vaccine Responses**<br>**Shane Crotty** La Jolla Institute for Immunology, USA
**18:30**	**WELCOME RECEPTION - POSTER SESSION I**

Day 2 Thursday 2 March

**Session E**	**Immunological Assays: Lessons from International Consortia**<br>**Chairs: Rebecca Jane Cox** University of Bergen, Norway **Paul Thomas** St Jude Children's Research Hospital, USA
**09:00**	**Lessons Learned from SARS CoV-2 Assays**<br>**David Montefiori** Duke University School of Medicine, USA **(remote)**
**09:30**	**Lessons Learned from FLUCOP: HA Antibody Assays**<br>**Othmar Engelhardt** Medicines and Healthcare Products Regulatory Agency (MHRA), UK
**10:05**	**Lessons Learned from FLUCOP: NA Antibody Assays**<br>**Emanuele Montomoli** University of Siena, Italy
**10:20**	**Lessons Learned from FLUCOP: T Cell Assays**<br>**Gwenn Waerlop** Ghent University, Belgium
**10:40**	**BREAK**
**Session F**	**Regulatory Session**<br>**Chairs: Jerry Weir** U.S Food and Drug Administration (FDA), USA **Chris Roberts** National Institutes of Health (NIH), USA
**11:10**	**Introduction to Regulatory Hurdles for Next Generation Influenza Vaccines**<br>**David Vaughn** Bill & Melinda Gates Foundation, USA<br><br>**Regulators' Perspective**<br>**Jerry Weir** Food and Drug Administration (FDA), USA **Marco Cavaleri** European Medicines Agency (EMA), Netherlands **[remote]**
**11:30**	**PANEL DISCUSSION: How Can Correlates of Protection Help the Regulatory Process for Licensure of New Vaccines?**<br>**Sarah Gilbert** University of Oxford, UK<br>**Chris Chiu** Imperial College London, UK<br>**Chris Roberts** National Institutes of Health (NIH), USA<br>**David Vaughn** Bill & Melinda Gates Foundation, USA<br>**Jerry Weir** Food and Drug Administration (FDA), USA<br>**Marco Cavaleri** European Medicines Agency (EMA), Netherlands [remote]

**12:30**	**LUNCH**
**Session G**	**Optimal Study Design for Establishing Correlates of Protection**<br>**Chairs: Joe Bresee** The Taskforce for Global Health, USA<br>**Sheena Sullivan** WHO Collaborating Centre for Reference and Research on Influenza, Peter Doherty Institute for Infection and Immunity, Australia
**13:45**	**Influenza Vaccine Effectiveness**<br>**Sheena Sullivan** WHO Collaborating Centre for Reference and Research on Influenza, Peter Doherty Institute for Infection and Immunity, Australia
**14:15**	**Novel Study Design to Support Correlates of Protection Studies**<br>**Peter B. Gilbert** University of Washington, USA
**14:45**	**Mathematical Modelling of Correlates of Protection**<br>**Miles Davenport** Kirby Institute, UNSW Sydney, Australia
**15:05**	**PANEL DISCUSSION – Study Design for Correlates of Protection**<br>**Aubree Gordon** University of Michigan, USA<br>**Miles Davenport** Kirby Institute, UNSW Sydney, Australia<br>**Sheena Sullivan** WHO Collaborating Centre for Reference and Research on Influenza, Peter Doherty Institute for Infection and Immunity, Australia<br>**Peter B. Gilbert** University of Washington, USA
**16:00**	**BREAK**

Session H
16:30-18:00

Oral Abstracts

Chairs: **S. Mark Tompkins** University of Georgia, USA **Emanuele Montomoli** University of Siena, Italy

Antibody titers and shedding dynamics of seasonal influenza infections in a South African community cohort (PHIRST), 2016-2017
Molly Sauter Princeton University, USA [ACOR0070]

Impact of COVID-19 vaccination on duration of viral shedding in asymptomatic and symptomatic infection
Nicole Ngai Yung Tsang WHO Collaborating Centre for Infectious Disease Epidemiology and Control, Hong Kong [ACOR0091]

Characterization of Viral Shedding and Particle Release from Humans Experimentally Infected with Seasonal H3N2 virus
Seema Lakdawala Emory University, USA [ACOR0096]

Trends in the age immunity profile of respiratory viruses throughout the COVID19 pandemic in Seattle, 2020-2022
Cecile Viboud Fogarty International Center, USA [ACOR0102]

Protective cross-reactive human monoclonal antibodies against influenza virus neuraminidases targeting the conserved catalytic site
Julia Lederhofer Vaccine Research Center/NIAID/NIH, USA [ACOR0033]

Mapping the differential adaptive immune dynamics to distinct influenza vaccine modalities using human tonsil organoids
Lisa Wagar University of California Irvine, USA [ACOR0097]

Baseline innate and T cell populations are correlates of protection against symptomatic influenza virus infection independent of serology
Robert Mettelman St. Jude Children's Research Hospital, USA [ACOR0069]

18:00 **EVENING RECEPTION - POSTER SESSION II**

DAY 3 Friday 3 March

**Session I** 09:00 – 10:30	**Oral Abstracts**<br>**Chairs:** **Chris Chiu** Imperial College London, UK **Stacey Schultz-Cherry** St. Jude Children's Research Hospital, USA
	**Distinct functional humoral immune responses are induced after live attenuated and inactivated seasonal influenza vaccination**<br>**Xin Tong** MIT and Harvard, USA [ACOR0001]<br><br>**Assessment of multiple immune correlates of protection against influenza using acute and convalescent sera from influenza natural infection**<br>**Min Levine** Centers for Disease Control and Prevention, USA [ACOR0040]<br><br>**Immune-history based correlates of protection for influenza and SARS-CoV-2**<br>**Tomer Hertz** Ben Gurion University of the Negev, Israel [ACOR0061]<br><br>**Post-vaccine cytokine levels that correlate with breakthrough influenza infections**<br>**Ewan P. Plant** Food and Drug Administration, USA [ACOR0002]<br><br>**Influenza vaccine responses to A(H1N1)pdm09 antigens in 2020 and 2021 among repeatedly vaccinated healthcare workers**<br>**Stephany Sanchez-Ovando** University of Melbourne, Australia [ACOR0027]<br><br>**Age-dependent induction of stalk-reactive antibodies with ADCC reporter activity by administration of a live-attenuated influenza virus vaccine to children**<br>**Juan Manuel Carreno Quiroz** Icahn School of Medicine at Mount Sinai, USA [ACOR0064]<br><br>**Pre-existing SARS-CoV-2 antibodies and risk of breakthrough infection in a prospective cohort, Seattle, March-November 2022**<br>**Alpana Waghmare** University of Washington, USA [ACOR0101]
**10:30**	**BREAK**

Session J 11:00-12:30	**Oral Abstracts**<br>**Chairs: Florian Krammer** Icahn School of Medicine at Mount Sinai, USA<br>**Diane Post** National Institute of Allergy and Infectious Diseases (NIAID), USA
	**Matrix M adjuvanted H5N1 vaccine elicits broadly neutralizing antibodies and neuraminidase inhibiting antibodies in humans that correlate with in vivo protection**<br>**Fan Zhou** University of Bergen, Norway [ACOR0019]<br><br>**Human influenza virus challenge identifies cellular correlates of protection for oral vaccination**<br>**David McIlwain** Stanford University, USA [ACOR0079]<br><br>**An influenza hemagglutinin stem-only Immunogen elicits a broadly cross-reactive memory B cell response in humans**<br>**Sarah Andrews** National Institutes of Health, USA [ACOR0039]<br><br>**SARS-CoV-2 correlates of protection conferred by natural immunity: comparative analysis of pre-exposure neutralizing antibody titers against Delta and Omicron variant infection**<br>**Kaiyuan Sun** National Institutes of Health, USA [ACOR0021]<br><br>**Detailed estimates of correlates of protection for SARS-CoV-2, stratified by key VOC and key covariates**<br>**Tim Russell** London School of Hygiene and Tropical Medicine, UK [ACOR0098]<br><br>**Antibody effector function and T cell responses to homologous and heterologous inactivated or mRNA vaccines against SARS-CoV-2**<br>**Carolyn Cohen** University of Hong Kong, Hong Kong [ACOR0013]
12:30	LUNCH

**Session K** 13:30-14:30	**Recap of Sessions A-D**
	**Group Discussion**<br>**Lessons Learned from COVID for Identifying Correlates of Protection and Developing Consensus on Immune Responses to Target**<br><br>**Florian Krammer** Icahn School of Medicine at Mount Sinai, USA<br>**Sarah Gilbert** University of Oxford, UK<br>**Rebecca Jane Cox** University of Bergen, Norway<br>**Kanta Subbarao** WHO Collaborating Centre for Reference and Research on Influenza, Peter Doherty Institute for Infection and Immunity, Australia<br>**Cheryl Cohen** University of Witwatersrand, South Africa
**14:30**	**BREAK**
**Session L** 15:00-16:00	**Recap of Sessions E-G**
	**Group Discussion**<br>**The Way Forward in Correlates of Protection and their Detection for Next Generation Vaccines**<br><br>**Jerry Weir** FDA, USA<br>**Jacqueline Katz** The Taskforce for Global Health, USA<br>**Othmar Engelhardt** Medicines and Healthcare Products Regulatory Agency (MHRA), UK<br>**Sheena Sullivan** WHO Collaborating Centre for Reference and Research on Influenza, Peter Doherty Institute for Infection and Immunity, Australia<br>**S. Mark Tompkins** University of Georgia, USA
**16:00**	**End of Meeting - Closing Remarks** **Ros Hollingsworth** Bill & Melinda Gates Foundation, USA
